# WHAT IS THE DIGITAL MATURITY OF LONG-TERM CARE HOMES IN ONTARIO? A SURVEY PROTOCOL

**DOI:** 10.1093/geroni/igac059.253

**Published:** 2022-12-20

**Authors:** Ramtin Hakimjavadi

**Affiliations:** University of Ottawa, Ottawa, Ontario, Canada

## Abstract

Ramtin Hakimjavadi will discuss the barriers and facilitators of implementing a health information technology (HIT) maturity survey in the Canadian context. Alongside Dr. Liddy’s team, he is supporting the adaptation of the survey into a Canadian context, followed by a pilot study to distribute the survey to select nursing homes. Drawing from this work, Mr. Hakimjavadi will highlight the barriers and facilitators associated with the process of implementing the HIT maturity survey. Expected challenges include adjusting the survey format for respondents with limited resources, identifying a central contact within each nursing home, optimizing the survey user interface to limit response burden, and developing recruitment strategies to increase response rate. These challenges and the strategies to overcome them, impressions of the current state of HIT in Ontario nursing homes, and proposed action items to trend digital maturity in these settings over time will be discussed.

